# The Poor Man's Home.—XXVIII

**Published:** 1898-03-05

**Authors:** 


### Modern Sociology.

### THE POOR MAN'S HOME.—XXVIII.

### Conclusion.

We have summarised in the preceding articles much of what has been done in improving the housing of the working classes. Much more might be said, but it would largely repeat what has been told already. Every year another town takes up the question, and in its treatment of it benefits by what has been done by others. It would, therefore, only weary the reader to detail the improvements accomplished or projected in almost every city in the kingdom, and in many foreign towns. But, in conclusion, we would like to summarise the good that may be expected to result from the improvement of the dwellings of the poor.

A critic, for whose opinion as a practical philanthropist the present writer has the highest respect, asked, after reading one of the earlier papers, if the ideas propounded in it were economically justifiable. The examples given in many of the articles which followed show that, at least, healthy houses for the less fortunate classes can be made remunerative investments, and, therefore, economically justifiable from the standard of the money market. This is a point of view which the present writer would be most unwilling to ignore, knowing that to give alms, either open or disguised, to the poor is, more often than not, to do them the greatest harm. But, this being admitted, we may carry our economics a step farther.

As a matter of economy, it is desirable, in the interests of both labourer and employer, that the work of the former should be as efficient as possible. Highly-paid labour is often more economical than cheap labour, just because it is more efficient. Whatever conduces to efficiency in labour, whether it be machinery or men, is a true economy. Now, good houses contribute largely to this economic efficiency. It is true that people live, and bring up children, in dens which outrage every dictate of sanitary science. But, in economics, not life so much as labour must be our test. What is the labour of these people worth? Not much, it will be admitted by those who have come in contact with them. A countryman, coming from a home, however poor, where he has been surrounded by pure air, will do, perhaps, twice as much. Yet the countryman would die in a month if he were put to live in the insanitary homes of the city worker; or, at the best, would gradually become less and less efficient in his work. Mere survival, though it may prove fitness for a given environment, does not of itself justify existence. That weeds will thrive in a soil that will not grow wheat is not regarded as a reason for allowing our land to lie desert, for the benefit of tares and thistles. Improve the soil, and many of the most noxious weeds will disappear spontaneously. Many of the inhabitants of the poorer quarters of our great cities are little more than cumberers of the ground. To provide more healthy houses may, indeed, do little for these, but it will do much to enable their children to rise to a higher state of industrial efficiency, and in any case will make it possible, as now it is not, in many places, to bring a worker from the country to the town, without injuring the quality of his work, or dooming him to an untimely death.

There is another economic test. We reckon our effective population, our labour army, not by the number of individuals who are born, but by those who survive to the working age, the age at which they begin to repay to the community the money that has been spent on rearing them. (This argument is not affected by the fact that the child when grown to the working age does not repay to his parents the cost of his previous maintenance. It is enough that he, by his labour, adds something to the total wealth of the community.) Every child born, who does not live to be a worker, is a loss to the community. Now, we have Dr. Russell's word for it that the vast majority of the children who die in Glasgow, under the age of five years, have spent their brief and sickly lives in one-room dwellings; that is, in dwellings which are overcrowded, and therefore insanitary. The same is more or less true of all our large towns. Each of those children is a loss to the community, a loss which becomes greater with every year that a child lives who yet dies before reaching the age of work. If a large mortality among children can be shown to result from unhealthy dwellings—as we think it can—there are the strongest economic reasons for building dwellings where life may be prolonged. For, be it remembered, people do not stop bringing children into the world because they live in unhealthy houses; they only fail to rear them. As a matter of fact, the very recklessness of those classes which inhabit the worst houses leads them to marry and bring children into the world in a way which the richer classes do not imitate partly because they have a clearer idea of parental responsibilities, and partly because their own personal needs are so much greater that they cannot afford to bring up a large family. Such people postpone marriage till they have an income to meet the expenses which marriage brings. But, as they have the power and the will to do the best possible for such children as they have, they contribute as much—perhaps more—to the labour strength of the country as those who give birth to a greater number of children, a large proportion of whom die before reaching maturity. Indeed, if intelligence and efficiency be taken into account, the few children of the prudent parent may contribute more to the community than the more numerous surviving offspring of the careless and improvident. A man who will not live in one room with a large and growing family may be a better citizen than he who does. The thing for the philanthropic individual or far-seeing community to do, is to give each citizen a chance of living in such conditions as will encourage him to add to its numbers only such a number of young citizens as he can bring up in decent comfort and rear to industrial efficiency.

If the community thus gives the poor man those conditions of health which are necessary for himself and his family, but which he could not attain without such help, the community has in turn a right to demand as rent such decency of life, such cleanliness of habit as will prevent him and his children being a danger either to the health or the morals of his neighbours. And if each party to the arrangement does well for itself while doing well for the other, as we believe both do, then we think there is the amplest economic justification for anything that can be done towards improving "The Poor Man's Home."

